# INCREASED PHYSIOLOGICAL VARIABILITY PREDICTS DECLINING HEALTH AND CRITICAL TRANSITIONS IN HEMODIALYSIS PATIENTS

**DOI:** 10.1093/geroni/igz038.2905

**Published:** 2019-11-08

**Authors:** Alan A Cohen, Yuichi Nakazato, Tomoko Sugiyama, Diana L Leung, Véronique Legault, Anne-Marie Côté

**Affiliations:** 1 University of Sherbrooke, Sherbrooke, Quebec, Canada; 2 Hakuyukai Medical Corporation, Saitama-shi, Japan; 3 Yale University, New Haven, Connecticut, United States

## Abstract

Increased variability in levels of several individual biomarkers has been shown to predict adverse outcomes, particularly in hemodialysis patients, for whom time series data is often available. Here, we evaluate the feasibility of using multivariate approaches to quantify global physiological variability as a potential predictor of adverse outcomes. We used data on 588 deaths and 1196 hospitalisations across ~38,000 visits of 591 hemodialysis patients at a Quebec hospital, as well as data on frailty and mortality in 580 patients assessed 20+ times within a one-year period at a hospital in Saitama, Japan. We use two approaches: principal components analysis (PCA) of the coefficients of variation (CVs) of the individual biomarkers over the previous year, and Mahalanobis distance (MD) of the biomarker profile relative to the same profile at the previous time point. We show that both methods provide substantial prediction of both impending mortality and impending hospitalisation, with hazard ratios across the 95% quantile range of the indices varying between 1.5 and 3.5 (p<0.0001). Each unit change on the first PCA axis (PC1) increased frailty odds by 2.34 (95% CI: 1.21-4.52). PCA performed substantially better than MD. CVs of various biomarkers were consistently positively correlated, and PC1 was a good predictor of frailty, mortality, and hospitalisation. Overall, these results confirm that complex physiological integration can break down, resulting in loss of homeostatic control and increasing variability, as predicted by complex systems theory. The resulting indices provide a predictive signal of impending critical health transitions, with both theoretical and clinical implications.

